# Alcohol consumption and its interaction with adiposity-associated genetic variants in relation to subsequent changes in waist circumference and body weight

**DOI:** 10.1186/s12937-017-0274-1

**Published:** 2017-08-25

**Authors:** Jeanett F Rohde, Lars Ängquist, Sofus C. Larsen, Janne S. Tolstrup, Lise Lotte N. Husemoen, Allan Linneberg, Ulla Toft, Kim Overvad, Jytte Halkjær, Anne Tjønneland, Torben Hansen, Oluf Pedersen, Thorkild I. A. Sørensen, Berit L Heitmann

**Affiliations:** 1 Research Unit for Dietary Studies at the Parker Institute, Bispebjerg and Frederiksberg Hospital, the Capital Region, Copenhagen, Nordre Fasanvej 57, entrance 5, ground floor, 2000 Frederiksberg, Denmark; 2 Department of Clinical Epidemiology (Formerly ‘Institute of Preventive Medicine’), Bispebjerg and Frederiksberg Hospital, the Capital Region, Nordre Fasanvej 57, Hovedvejen, entrance 5, first floor, 2000 Frederiksberg, Denmark; 3 National Institute of Public Health, University of Southern Denmark, Øster Farimagsgade 5 A, 1353 Copenhagen K, Denmark; 4 Research Centre for Prevention and Health, Capital Region of Denmark, Nordre Ringvej 57, building 84-85, 2600 Glostrup, Denmark; 5 Department of Clinical Experimental Research, Rigshospitalet, Nordre Ringvej 57, 2600 Glostrup, Denmark; 6 Department of Clinical Medicine, Faculty of Health and Medical Sciences, University of Copenhagen, Blegdamsvej 3B, 2200 København N, Denmark; 7 Section for Epidemiology, Department of Public Health, Aarhus University, Nordre Ringgade 1, 8000 Aarhus C, Denmark; 8 Department of Cardiology, Aalborg University Hospital, Fredrik Bajers Vej 7-D3, 9220 Aalborg, Denmark; 9 Danish Cancer Society Research Center, Strandboulevarden 49, 2100 Copenhagen Ø, Denmark; 10 The Novo Nordisk Foundation Center for Basic Metabolic Research (Section of Metabolic Genetics), and Department of Public Health, Faculty of Health and Medical Sciences, University of Copenhagen, Nørre Alle 20, 2200 Copenhagen N, Denmark; 11 MRC Integrative Epidemiology Unit, Bristol University, Senate House, Tyndall Avenue, Bristol, BS8 1TH UK; 12 The Boden Institute of Obesity, Nutrition, Exercise & Eating Disorders, The University of Sydney, Sydney, NSW 2006 Australia; 13 Section for General Practice, Department of Public Health, University of Copenhagen, Øster Farimagsgade 5, entrance Q, 1014 Copenhagen K, Denmark

**Keywords:** Alcohol, Weight change, Waist change, Genetic predisposition, SNP score, Gene-diet interaction

## Abstract

**Background:**

Studies have suggested a link between alcohol intake and adiposity. However, results from longitudinal studies have been inconsistent, and a possible interaction with genetic predisposition to adiposity measures has often not been taken into account.

**Objective:**

To examine the association between alcohol intake recorded at baseline and subsequent annual changes in body weight (∆BW), waist circumference (ΔWC) and WC adjusted for BMI (ΔWC_BMI_), and to test for interaction with genetic predisposition scores based on single nucleotide polymorphisms (SNPs) associated with various forms of adiposity.

**Method:**

This study included a total of 7028 adult men and women from *MONICA*, the Diet, Cancer and Health cohort (*DCH*), and the *Inter99* studies. We combined 50 adiposity-associated SNPs into four scores indicating genetic predisposition to BMI, WC, WHR_BMI_ and all three traits combined. Linear regression was used to examine the association of alcohol intake (drinks of 12 g (g) alcohol/day) with ΔBW, ΔWC, and ΔWC_BMI,_ and to examine possible interactions with SNP-scores. Results from the analyses of the individual cohorts were combined in meta-analyses.

**Results:**

Each additional drink/day was associated with a ΔBW/year of −18.0 g (95% confidence interval (CI): −33.4, −2.6, *P* = 0.02) and a ΔWC of −0.3 mm/year (−0.5, −0.0, *P* = 0.03). In analyses of women only, alcohol intake was associated with a higher ΔWC_BMI_ of 0.5 mm/year (0.2, 0.9, *P* = 0.002) per drink/day. Overall, we found no statistically significant interactions between the four SNP-scores and alcohol intake in relation to changes in adiposity measures. However in analyses of women separately, we found interaction between the complete score of all 50 SNPs and alcohol intake in relation to ΔBW (P for interaction = 0.03). No significant interaction was observed among the men.

**Conclusion:**

Alcohol intake was associated with a decrease in BW and WC among men and women, and an increase in WC_BMI_ among women only_._ We found no strong indication that these associations depend on a genetic predisposition to adiposity.

**Trial registration:**

Registry: ClinicalTrials.gov Trial number: CT00289237, Registered: 19 September 2005 retrospectively registered.

**Electronic supplementary material:**

The online version of this article (10.1186/s12937-017-0274-1) contains supplementary material, which is available to authorized users.

## Background

Alcoholic beverages are energy dense and often an addition to the total daily energy intake from other  macronutrients. These extra calories from alcohol may contribute to weight gain. A cross-sectional study by Tolstrup et al. 2005 [1] showed that total alcohol intake among men and women was directly associated with high body mass index (BMI) (≥30 kg/m^2^) and large waist circumference (WC) (≥102 cm for men and ≥88 cm for women) [1]. The same study also showed that among both men and women, frequent drinking was associated with the lowest odds of being overweight and having an increased WC for given level of total alcohol intake [1]. Another study, using follow-up data from the same cohort, found that drinking frequency was inversely associated with subsequent major waist gain, and not associated with major waist loss [2]. Alcohol consumption has also shown to be a contributing factor to an increase in body weight, body fat and BMI among Korean women [3]. However, the majority of cross-sectional studies published since 2005 indicates no association between light to moderate alcohol intake and obesity [4]. Adding to the complexity, studies have found that type of alcohol may affect weight differently; thus, intake of wine may protect against weight gain, whereas intake of spirits and beer may be directly associated with weight gain, but this still needs confirmation [4–6].

The inconsistent evidence relating alcohol intake to the development of obesity may be the product of not taking potential confounding factors or effect modifiers into account, such as gender, frequency and amount of alcohol, sleeping habits, physical activity, disease and history of alcohol use [4, 5]. Smoking is also strongly associated with alcohol intake [7] and may have a confounding effect on the relationship between alcohol intake and body weight changes. However, also genetic influences may be involved in these associations. Indeed, Corella et al. 2012 examining the interaction of variants of the MC4R and FTO genes with various dietary factors including alcohol consumption on obesity did not find evidence for interactions with alcohol, but they found lower alcohol consumption in people carrying the variant alleles [8]. Furthermore, Greenfield et al. 2003 [9] in a co-twin case-control study including 334 female twins, showed an inverse relationship between alcohol consumption and total and abdominal fat, independently of known environmental confounders. However, they also found that the association between moderate alcohol consumption and abdominal, but not total fat was dependent on genetic risk; those genetically predisposed appeared protected against abdominal fat accumulation. In these individuals, a daily intake of 1–1.5 alcoholic drinks was associated with approximately 20% less abdominal fat than among individuals of a similar genetic risk with alcohol intakes of less than one drink per week [9].

On this background, we aimed to investigate the association between alcohol intake and changes in body weight (BW) (ΔBW; g/year), in WC (ΔWC; mm/year), and in WC adjusted for given BMI (ΔWC_BMI_; mm/year), over a period of 5 years, while taking relevant confounding factors and possible interaction with gender into account. We further aimed to investigate the possible interaction between alcohol intake and molecular genetic predisposition, assessed by scores based on single nucleotide polymorphisms (SNPs), associated to various forms of adiposity, in relation to ΔBW, ΔWC or ΔWC_BMI_.

## Methods

Information for this study was obtained from three different Danish cohorts of adults; The *MONICA*, the *Inter99* and the *Diet, Cancer and Health (DCH)* studies, with no possibility of overlapping participants. Furthermore, all participants had information on alcohol intake and subsequent changes in anthropometric measurements. Also information on SNPs associated with different measures of adiposity, as well as information on potential confounders was available. In total, 7028 participants were included in the present study.

### The cohorts

#### Monica

The *MONICA* (MONItoring trends and determinants of CArdiovascular disease) study consisted of 4581 men and women born in 1922,1932,1942 and 1952 from 11 municipalities around the former Copenhagen County. Of these, 3608 participated in a baseline health examination during 1982–1983 and 5 years later during 1987–1988. In total, 2987 men and women chose to participate in the second health examination [10]. Between 1982 and 1983, a total of 1852 participants completed a 7-day dietary record [11]. Moreover, 1578 participants had information on relevant covariates and repeated measurements on body weight, and 1426 participants had information on genetic variants. Participants with prevalent cancer (*n* = 16), cardiovascular disease (*n* = 61) or diabetes (*n* = 20) at baseline were excluded from this study. Furthermore, participants with incident cancer (*n* = 13), cardiovascular disease (*n* = 57) or self-reported diabetes (*n* = 2) during follow-up were also excluded. The final cohort-population consisted of 1257 participants.

#### Inter99

*Inter99* was a randomised multifactorial lifestyle intervention study (CT00289237, ClinicalTrials.gov), focusing on prevention of ischemic heart disease through repeated lifestyle counselling. Short-term outcomes included changes in lifestyle factors, plasma cholesterol, blood pressure and body mass index. The study was conducted in 11 municipalities around the former Copenhagen County. The study group was a randomly selected age- and gender-stratified sample of 13,016 people born between 1939 and 1940, 1944–1945, 1949–1950, 1954–1955, 1959–1960, 1964–1965 and 1969–1970 and were all invited to a baseline health examination during 1999–2001. A total of 6784 accepted to participate in the baseline examination, where blood samples and physical tests were taken. Moreover, self-administered questionnaires regarding health status and validated 198-item food frequency questionnaires (FFQ) [12] were filled out. Participants were invited to participate in a follow-up examination during 2004–2006 [13]. In total, 4544 participants had information from baseline and follow-up examination on diet, genes, anthropometric measures and information on potential confounders. The study, and each intervention component, has been described in detail elsewhere [14, 15].

For this study, participants with prevalent cancer (*n* = 87), cardiovascular disease (*n* = 318) or self-reported diabetes (*n* = 93) at baseline, and participants with incident cancer (*n* = 68), cardiovascular disease (*n* = 254) or self-reported diabetes (*n* = 118) were excluded. The final cohort population consisted of 3606 participants.

#### Diet, cancer and health study

The *DCH* study was based on a total of 160,725 men and women, with no diagnosis of cancer in the Danish Cancer Register, from the Aarhus and Copenhagen area that were invited to participate in a baseline examination during 1993–1997 and a follow-up examination during 1999–2000 [16]. In total 57,053 accepted the invitation. All participants filled in a lifestyle questionnaire and a 192 item semi-quantitative FFQ and had their anthropometric measurements taken. For the DiOGenes study, a sub-cohort was selected from the *DCH* Study, including only the individuals who were younger than 60 yrs. at baseline and younger than 65 yrs. at follow-up in order to avoid the aging-related sarcopenia. Moreover, participants diagnosed with cancer, cardiovascular disease or diabetes at baseline and those who developed these diseases during follow-up were excluded. Participants were only included if they had stable smoking habits and had an average BW gain of no more than 5 kg/year during baseline and follow-up. A subsample of 2409 participants was selected, and it comprised 1200 (600 men and 600 women) body weight gainers, and 1209 randomly selected participants as controls. Body weight gainers were defined as those in the sub-cohort selected for DiOGenes who experienced the greatest annual weight gain during the follow-up period, and they were identified by using the residuals from a gender-specific regression model of annual BW change on baseline values of age, BW, height, smoking status and follow-up time. The randomly selected participants included 79 participants who were also defined as weight gainers, and they were excluded from the analyses (*n* = 1130) [17]. In total, 2165 had information on genes, alcohol intake covariates and ΔBW (2128 in analyses of ΔWC). However, 278 of these only had genetic information on FTO (rs9939609). The final cohort population therefore consisted of up to 2165 participants.

### Anthropometric measurements

In all three cohorts, baseline height and BW were measured to the nearest 0.5 cm for height and 0.1 kg for weight. Due to missing information on WC at baseline in the *MONICA* study, this study was not included in the analyses on changes in WC. In both the *Inter99* and the *DCH* cohort, baseline WC was measured to the nearest 1 cm, horizontally midway between the lower rib margin and iliac crest. The same procedures were used at follow-up. However, in the *DCH* the measurements of BW and WC (measured at the level of the umbilicus) were self-reported, but instructions were given on how to carry out the measurements combined with a paper measuring tape. A validation study was conducted in the *DCH* study to compare measurements of self-reported WC at the level of umbilicus and technician-measured natural WC and Spearman correlation coefficients of 0.88 in women and 0.87 in men were found [18]. Moreover, WC_BMI_ was calculated, defined as residuals of WC regressed on BMI (gender- and study-specific regressions; separately for baseline and follow-up values).

This measure was used instead of waist-hip ratio adjusted for BMI because no information on hip circumference was available at follow-up. The baseline correlation between waist-hip ratio adjusted for BMI and WC_BMI_ was 0.77 in *DCH* and 0.68 in *Inter99*. Due to different follow-up time in the three cohorts, and between the individuals in each cohort, average changes per year in BW, WC and WC_BMI_ was calculated as the difference between baseline and follow-up divided by the follow-up time in years for each individual.

### Assessment of alcohol intake

In all the cohorts, alcohol intake was recorded at baseline. In the *MONICA* study, intake of food and beverages was obtained by a 7-day dietary record and was completed within a 3 week period. Participants were given verbal and written instructions on how to record their diet. The individual average daily intake of macronutrients and total energy intake was calculated using the software Dankost (http://dankost.dk/). For the *Inter99* and *DCH* cohorts, FFQs were used to obtain information on diet and beverage intakes; very similar FFQs were used in both cohorts. The FFQs consisted of 198 and 192 items, respectively, and referred to the average intake of different foods and beverages within the last year. The individual average daily intake of macronutrients and total energy intake were calculated using the software FoodCalc *(* http://www.ibt.ku.dk/jesper/foodcalc/). Both Dankost and FoodCalc are based on the official Danish food-composition tables (http://frida.fooddata.dk/). The development and validation of the dietary records and FFQs used in the three cohorts has been described in detail elsewhere [12, 19, 20]. Alcohol intake was recorded as the average daily intake of beer, wine, dessert wine, and spirits within the last 12 months [21, 22]. From this information, we calculated daily intake of alcohol, reported as number of standard drinks (which in Denmark is defined as 12 g alcohol) per day, and total daily energy intake. Both alcohol consumption and total energy intake were included in the analysis as continuous variables.

### Possible confounders

Information on physical activity (PA) was self-reported in all three cohorts. In the *MONICA* study, participants were asked to classify themselves into one out of four categories: 1) Almost completely inactive, 2) Some physical activity, 3) Regular to hard activity, and 4) Hard activity. The validated Cambridge Physical Activity Index was used to assess PA in the *DCH* cohort [23] and was also categorised into four groups: 1) Inactive, 2) Moderately inactive, 3) Moderately active and 4) Active. In the *Inter99* study, information on time spent commuting and leisure time PA was used to categorize participants into four groups as: 1) 0- < 2 h/week, 2) 2- < 4 h/week, 3) 4- < 7 h/week and 4) ≥7 h/week. All participants had reported smoking habits and information was categorised into five groups: 1) Never smoked, 2) Former smokers, 3) tobacco 0- < 15 g/day, 4) tobacco 15- < 25 g/day and 5) tobacco ≥25 g/day. All participants gave information on years of regular schooling and this information was categorized as having school education above or below the primary level. Finally, information on age, gender, baseline height, menopausal status in women and total energy intake was available in all cohorts.

### SNP selection and genotyping

Based on a review of genome-wide association studies (GWAS) until 2010, 58 SNPs were identified to be associated with BMI, WC or WHR_BMI_ [24–36] and 50 of these SNPs were available in all 3 cohorts (Additional file 1). SNPs have not been investigated for their associations with WC_BMI,_ but the SNPs associated with WHR_BMI_ were considered pertinent to the WC_BMI,_ phenotype. Both in the *MONICA* and *DCH* cohort the SNPs were genotyped using the KASPar SNP genotyping method (KBioscience, Hoddesdon,UK) and the average genotyping success rates were respectively 98.3% in the *MONICA* cohort and 97.8% in the *DCH* cohort; 185 replicate samples in the *DCH* cohort had a success rate above 98% and an error rate below 0.5%. The SNPs were also successfully genotyped in the *Inter99* study using either the KASPar SNP genotyping method, or through Human Cardio-metabo bead chip array [37] with an average success rate of 96.7%.

### Genetic predisposition score

For every individual, the 50 SNPs were recoded into 0/1/2 according to number of obesity-associated risk alleles. Furthermore, genetic predisposition for each individual was defined through four SNP-scores and, in addition to the total score consisting all 50 SNPs, three phenotype-specific scores were created [38]. These SNP-scores have been shown to be associated with their concurrent phenotypes in our cohorts [38]. Scores were based on summing up the number of risk alleles with respect to contributing SNPs, a BMI score was based on 33 SNPs, a WC score on 6 SNPs, and the WHR_BMI_ score on 14 SNPs. Some of the scores were slightly overlapping with respect to sets of SNPs (Additional file 1). By construction, a higher score indicated an estimated greater predisposition to general adiposity and to the three phenotypes, respectively.

### Statistical analyses

Multiple linear regression models were used to examine the association between alcohol intake and changes in BW, WC and WC_BMI_ with adjustments for baseline measure of outcome, age, gender, height, smoking status, education, PA, and menopausal status. WC_BMI_ was additionally adjusted for baseline BMI. Males were coded as a separate menopausal group and the software took care of this inherited dependence (between gender and menopausal status) in the analysis while all men still remained in the analyzed sample.

We performed analyses both with and without adjustment for total energy intake. Inclusion of total energy in the model introduces a substitution model, implying conditioning on total energy, where a higher alcohol intake must be followed by a lower intake of energy from other non-specified sources. In the analyses without total energy intake, alcohol intake may in this sense vary without concomitant differences in the diet. Since no substantial difference was seen in the results from the two models, only the energy-adjusted results are presented.

Moreover, to examine whether genetic predisposition modifies the association between alcohol intake and changes in BW, WC and WC_BMI_, statistical interaction was investigated by adding SNP-score and SNP-score × alcohol variables to the model. Additionally, similar interaction analyses based on single SNPs and alcohol intake with adjustment for the same set of confounders as mentioned earlier were also performed.

Finally, we tested for gender interactions by adding product terms to the models (2-way interactions: alcohol × gender; and 3-way interactions: SNP-score × alcohol × gender). Significant interactions were further evaluated through stratified analyses.

After performing the analyses in each cohort, a fixed-effects meta-analysis, based on inverse variance-weighting, was performed in order to combine results from the three cohorts.

All analyses were performed using Stata 12.1 (StataCorp LP, College Station, Texas; www.stata.com) and *p*-values of ≤0.05 were considered as statistically significant. However, adjustment for multiple testing using the Bonferroni method was performed for the interaction analyses based on single SNPs and in the interaction analyses based on gender.

#### Sensitivity analyses

To check the robustness of the results, a number of sensitivity analyses were performed. We included baseline levels of the anthropometric measures in the primary models to avoid confounding or regression towards the mean. However, it has been suggested that adjustment for baseline values in analyses of changes can cause bias [39]. Thus, supplementary analyses were conducted without any adjustments for baseline measures of outcome variables. Finally, analyses were performed demanding stable smoking habits, i.e. by excluding individuals with non-stable smoking habits. Smoking stability was here indicated through belonging to the same smoking category both at baseline and at follow-up, where three categories were defined: 1) Never smokers, 2) Ex-smokers/occasional smokers and 3) Current smokers.

## Results

In total, 7028 participants were included in the present study, with 1257, 2165 and 3606 individuals from the *MONICA*, *DCH* and *Inter99* studies, respectively. Characteristics of the participants, together with information on alcohol intake, total energy intake, anthropometric measurements, SNP scores and covariates are given in Table 1. The table shows that subjects from the *MONICA and DCH* cohorts had the highest median alcohol intake (1.0 drink/day [5th–95th range: 0.0, 5.0] and 1.0 drink/day [0.0, 5.4] respectively). The lowest intake of alcohol was seen in the *Inter99* cohort with a median intake of 0.8 (0.0, 4.3). Participants in the *DCH* cohort showed the highest ΔBW with a median of 1.0 kg/year (1.1, 2.4) and ΔWC with a median of 1.4 cm (−0.8, 4.4). The genetic predisposition scores were nearly identical in the three cohorts in term of median and 5th/95th percentiles.

**Table 1:** Information on alcohol intake, anthropometric measurements, genetic predisposition scores and covariates^1^

	MONICA^1^	DCH	Inter99
*N*	*1257*	*2165*	*3606*
*Follow-up time (years)*	5.0 (4.9; 5.3)	*5.3 (5.0; 5.8)*	*5.4 (5.1; 5.7)*
*Dietary variables*			
Alcohol intake (drinks/day)	1.0 (0.0; 5.0)	1.0 (0.0; 5.4)	0.8 (0.0; 4.3)
Energy (MJ/d)	9.0 (5.1; 14.9)	8.8 (5.3; 14.3)	9.4 (5.2; 15.8)
*BW (kg)*			
Baseline (kg)	69.0 (52.0; 93.0)	77.1 (56.8; 104.4)	75.2 (54.9; 102.4)
Follow-up (kg)	69.9 (51.4; 94.7)	82.0 (58.0; 110.0)	76.0 (55.5; 103.5)
ΔBW (kg/year)	0.2 (−1.0; 1.6)	1.0 (−1.1; 2.4)	0.2 (−1.4; 1.7)
*WC (cm)*			
Baseline (cm)	-	90.0 (70.0; 112.0)	84.0 (67.0; 105.0)
Follow-up (cm)	-	98.0 (76.0; 121.0)	87.0 (69.0; 109.0)
ΔWC (cm/year)	-	1.4 (−0.8; 4.4)	0.5 (−1.3; 2.5)
*WC*_*BMI*_ *(cm)*			
Baseline (cm)	*-*	−0.3 (−7.9; 9.1)	−0.2 (−7.7; 8.5)
Follow-up (cm)	*-*	−0.2 (−11.5; 12.6)	−0.2 (−8.2; 9.1)
ΔWC_BMI_ (cm/year)	*-*	−0.0 (−2.0; 2.2)	0.0 (−1.4; 1.2)
*SNP-based variables* ^*2*^			
BMI score	29 (23;35)	28 (23; 35)	29 (23; 34)
WC score	3 (1; 6)	3 (1; 6)	3 (1; 6)
WHR_BMI_ score	14 (10; 18)	14 (10; 18)	14 (10; 18)
Complete score	44 (37; 52)	44 (37; 51)	44 (36; 51)
*Covariates*			
Age, baseline (years)	41.4 (30.6; 61.1)	53.0 (50.0; 58.0)	45.0 (34.7; 59.8)
Height (cm)	169.0 (156.0; 184.0)	171.0 (157.5; 186.0)	172.0 (158.0; 187.5)
Gender, female (%)	52.1	49.4	51.4
Smoking, never smokers (%)	30.1	41.4	40.8
Education, ≤primary school (%)	34.5	30.2	24.9
Physical activity, most sedentary group (%)	21.6	9.5	11.2
Menopausal status, post-menopausal (%)	41.7	55.6	25.9

Abbreviation: Body Mass Index (*BMI*), Body Weight (*BW*), Waist Circumference (*WC*), Waist-Hip-Ratio (*WHR*)

^1^Results presented as median (5–95 percentiles) unless otherwise stated

^1^Information on WC was not available in the MONICA cohort

^2^Sum of BMI, WC or WHR associated risk alleles. In MONICA: *n* = 941 on BMI score, *n* = 1185 on WC score, *n* = 1121 on WHR-score, *n* = 836 on complete score. In DCH: *n* = 1438 on BMI score, *n* = 1805 on WC score, *n* = 1624 on WHR-score, *n* = 1247 on complete score. In Inter99: *n* = 2211 on BMI-score, *n* = 2995 on WC score, *n* = 2903 on WHR score, *n* = 1837 on complete score

Information on the included SNPs, together with information on which obesity traits they have been found to be associated with are presented in Additional file 1.

**Fig. 1:**
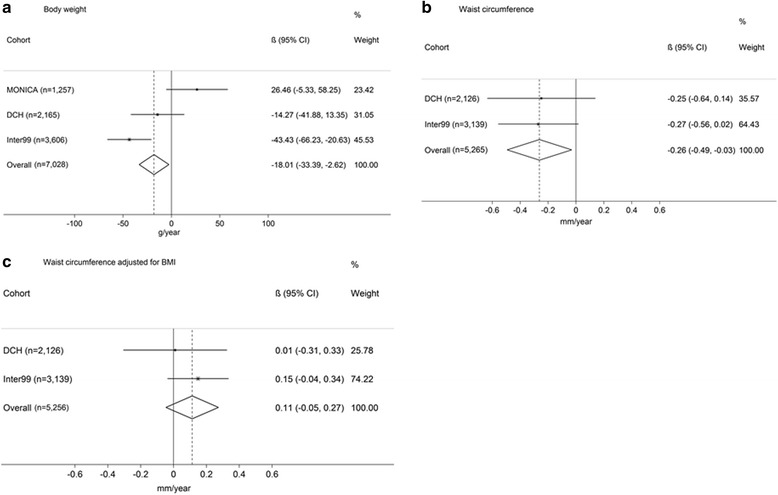
Annual change in BW, WC and WC_BMI_^1^ per 1 alcohol unit/day increase in alcohol intake. Results presented in g/year and mm/year, respectively. Model adjusted for baseline measure of outcome, age, gender, height, smoking status, education, physical activity, menopausal status and total energy intake.^1^Furthermore, case C is also adjusted for baseline BMI

The associations between alcohol intake and ΔBW, ΔWC and ΔWC_BMI_ are presented in Fig. 1. Results from the meta-analyses showed an inverse relationship between alcohol intake and ΔBW (−18.0 g per drink/day, [−33.4,-2.6], *P* = 0.02) (Fig. 1a). We also found an inverse association between alcohol intake and ΔWC (−0.26 mm per drink/day, [−0.5, −0.0], *P* = 0.03) (Fig. 1b). No significant association was seen for ΔWC_BMI_ (0.1 mm per drink/day, [−0.1, 0.3], *P* = 0.17) (Fig. 1c).

**Fig. 2:**
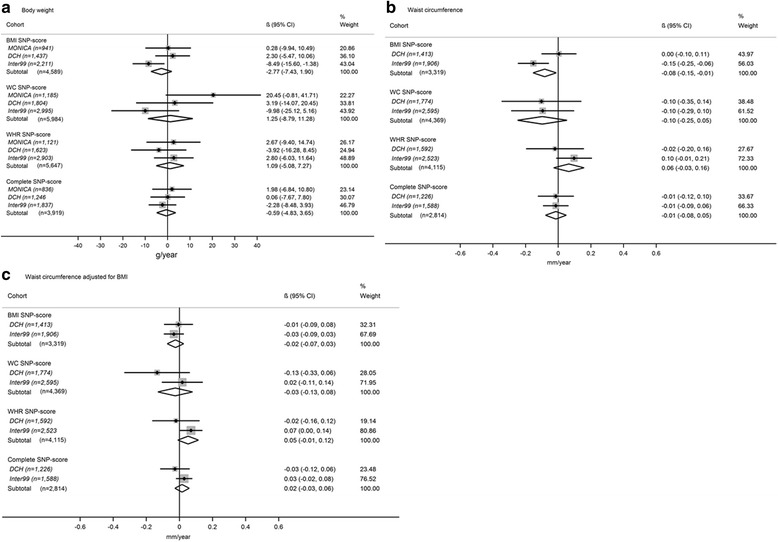
SNP score × alcohol interactions in relation to annual change in BW, WC and WC_BMI._^1^ Results presented in g/year and mm/year, respectively, and relate to the interaction (effect-modification) effects per additional risk allele for each alcohol unit/day. Model adjusted for baseline measure of outcome age, gender, height, smoking status, education, physical activity, menopausal status and total energy intake. ^1^Furthermore, case C is also adjusted for baseline BMI

Interactions between the four SNP-scores and alcohol intake in relation to ΔBW, ΔWC and ΔWC_BMI_ are presented in Fig. 2. Overall, the meta-analyses showed no interactions for any of the SNP-scores in relation to ΔBW, ΔWC or ΔWC_BMI._

Furthermore, after multiple testing, no significant results were seen in any of the interaction analyses based on individual SNPs in relation to ΔBW, ΔWC or ΔWC_BMI_ (Additional files 2, 3 and 4).

We found no significant interactions between gender and alcohol intake in relation to ΔBW or ΔWC. However, we did find an interaction between gender and alcohol in relation to ΔWC_BMI_ (*P* = 0.01). In stratified analyses, we found that alcohol was associated with a higher ΔWC_BMI_ among women (0.5 mm per drink/day, [0.2, 0.9], *P* = 0.002), but not among men (−0.03 mm per drink/day, [−0.2, 0.1], *P* = 0.75). In addition, we found a significant 3-way interaction between the complete score of all 50 SNPs, alcohol and gender in relation to ΔBW (*P* = 0.006). For women, each additional risk allele from the complete score was associated with a ΔBW of −10.2 g (−19.3, −1.1, *P* = 0.03) per drink of alcohol/day, while no significant interaction was observed among the men*.* When evaluating interaction effects from the individual SNPs included in the complete score, we found nominal significant interactions between alcohol and SFRS10 (rs7647305), DNM3-PIGC (rs1011731) and TNNI3K (rs1514175) among women, suggesting that each additional risk allele from these variants was associated with a lower ΔBW. For rs7647305, each additional risk allele was associated with a ΔBW of −86 g per drink/day (95% CI: -143, −29, *P* = 0.003), while each additional rs1011731 risk allele was associated with a ΔBW of −62 g per drink/day (95% CI: -108, −16, *P* = 0.008) and each additional rs1514175 risk allele was associated with a ΔBW of −52 g per drink/day (95% CI: -98, −5, *P* = 0.03). None of these variants showed significant interaction with alcohol among the men. Finally, we observed a significant 3-way interaction between the WHR_BMI_ score, alcohol and gender in relation to ΔBW (*P* = 0.04). However, in stratified analyses we found no significant WHR_BMI_-score × alcohol interaction for men or women. Moreover, using multiple testing correction, based on the Bonferroni-adjustment in the analysis on gender interaction, lead to that no results remained significant after such corrections.

Sensitivity analyses conducted without adjustment for baseline measure of outcome, and with respect to exclusions of individuals with non-stable smoking habits, did not notably change the observed associations (Additional files 5, 6, 7 and 8).

## Discussion

The present study was designed in order to investigate the association between alcohol intake and ΔBW, ΔWC and ΔWC_BMI_ and to examine possible interaction with genetic predisposition to different measures of adiposity. In the meta-analyses of the three cohorts, we found a statistically significant inverse association between alcohol intake and ΔBW and ΔWC. Furthermore, alcohol intake was associated with a higher ΔWC_BMI,_ but only among women. No statistically significant interactions were seen between alcohol and SNP-scores or with any individual SNPs. However, in analyses of women separately, we found interaction between the complete score of all 50 SNPs and alcohol intake in relation to ΔBW. The confidence intervals were narrow, making it unlikely that notable interactions were overlooked.

The majority of previous cross-sectional studies indicated no association between light to moderate alcohol intake and obesity [4]. In addition, evidence from longitudinal studies are inconsistent, and several studies have found no association or inverse associations between alcohol intake and BW change [4]. Similarly, our study showed inverse associations between alcohol intake and both ΔBW and ΔWC. This study further revealed that alcohol intake was associated with a higher ΔWC_BMI_ among women, which is in line with the findings reported by Hee-Ju et al. showing that alcohol consumption was associated with an increase in body weight, body fat and BMI among Korean women [3].

These findings were seen both with and without adjustment for total energy, suggesting no mediation from total energy on the relationship between alcohol intake and weight development.

The number of previous studies on gene × alcohol interaction and adiposity is limited. In the study by Greenfield et al. 2003 [9], an interaction analysis suggested that women with a predisposition to abdominal obesity were protected by a moderate alcohol intake [9]. In line with this, results from our interaction analyses suggested that an interaction between the complete score of all 50 SNPs and alcohol may be present among women, only. Our results are to some extent also in line with previously reported results suggesting sexually dimorphic associations between a number of known variants and anthropometric traits [40], but whether or not the interaction with alcohol intake truly is gender-specific needs further investigation. Furthermore, as we found some evidence that SFRS10 (rs7647305), DNM3-PIGC (rs1011731) and TNNI3K (rs1514175) may interact with alcohol in relation to ΔBW among women only, these specific variants may be worth investigating further in other cohorts. However, we did not adjust our main analyses for multiple testing, and we cannot exclude that this is a false positive result. Indeed, with an unadjusted *p*-value of 0.03, this interaction would not remain statistically significant when adjusting for the number of included SNP scores (i.e. unadjusted *p*-value of 0.03 × 4 SNP scores = 0.12).

The strengths of this study include the use of data from three large cohorts with detailed questionnaire information on alcohol consumption and energy intake, repeated measures of anthropometry, as well as information on several potential confounders including detailed information on participants’ smoking habits. Also, we had information on 50 genetic variants found previously to be consistently associated with BMI, WC or WHR_BMI,_ at genome-wide significance levels, making it possible to calculate genetic risk scores. Furthermore, we conducted prospective analyses, thereby limiting the influence of reverse causality.

Our study also has some limitations. Although the genetic risk scores were based on information from many established BMI, WC and WHR_BMI_ associated SNPs, these variants only explain a limited proportion of the total variation in obesity (<2%). Also, the SNPs included in this paper were identified through review of GWAS published until 2010 [24–36]. Since then, several additional variants have been identified. However, the newly identified SNPs generally explain an even smaller proportion of the variation in BMI, than those identified in the first rounds of GWAS [41, 42], Thus, if interactions between the genetic risk scores and alcohol intake in relation to change in weight and waist circumference depends on how much of the total genetic predisposition to obesity these explain, we would not expect additional SNPs to add much to the current results.

It may be considered to investigate other genetic variants than those known to be associated with BMI and WC, such as variants affecting insulin sensitivity or fatty liver disease, which, may be bi-directionally implicated in the changes over time in BW and WC. However, such data was available only for some of the cohorts in the present study and hence analyses would be performed with sub-optimal power.

Moreover, while the included SNPs associate with the level of adiposity or fat distribution, they do not specifically associate with changes in the adiposity measures, and it is possible that another so far unidentified set of SNPs, specifically related to gain in general and abdominal obesity, may show different results. Indeed, we showed previously that although these genetic risk scores were strongly associated with BW and WC in cross sectional designs, they were not generally associated with changes over time in BW or WC [38]. Also, although we included information on up to 7028 participants, it is possible true associations were still missed due to a lack of statistical power. Yet, the generally quite small effect-estimates and narrow CIs suggest that it is unlikely we have overlooked associations with any public health relevance. The dietary information on alcohol intake could be subject to misclassification. Non-differential misclassification could have weakened the associations observed, but if some kind of selective under-reporting occurred, it could have led to spurious findings. Nevertheless, self-reported measures of alcohol intake show reasonable levels of reliability and validity [43]. In view of the small size of Denmark, and the homogeneity of its population, it is very unlikely that there were any geographic differences, which potentially could have affected our outcomes, since all participants came from either the greater Copenhagen area (the capital of Denmark) or Aarhus city (Second largest city in Denmark). However, study heterogeneity could be a problem if studies outside Denmark were included.

Missing information on which type of alcohol that may have been responsible for the observed inverse association and the observed gender interaction is a potential limitation of the present study. However, investigating the interaction between type of alcohol intake and molecular genetic predisposition to adiposity in relation to subsequent changes in waist circumference and body weight is beyond the scope of the present paper, but could be a relevant aim for future studies, if additional cohorts including the pertinent information could be identified. Although we adjusted for many potential confounders, we cannot exclude the possibility of unmeasured or residual confounding potentially affecting our results. In this context, smoking habits are closely linked to both BW regulation and alcohol intake and in addition to baseline adjustment for smoking habits, we performed sensitivity analyses while excluding individuals with non-stable smoking habits and this did not change the observed associations. Furthermore, we did not exclude participants that were undergoing thyroid hormone therapy and hormone replacement therapy, however, we adjusted for menopausal status and since we expect this group to be a very small proportion of the entire study population of otherwise healthy individuals in general we do not think this would change the observed associations.

We cannot rule out whether underlying disease has affected our results, although through information from Danish health registers it was possible for us to exclude participants who had a chronic disease at baseline. In order to ensure that the results were not affected by diseases not yet diagnosed at baseline, we also excluded participants who developed a chronic disease during the follow-up period. Again results were essentially similar before and after this exclusion. Another limitation in the current study may be that the included cohorts and the study design were different. In general, no major differences were seen between the cohorts in relation to the main results, i.e. the associations between exposure and outcome, indicating that the other differences had little impact on the results. It may be argued that, given the diversity of study designs, combining the results of the three studies in a meta-analysis may be misleading. However, our analyses did not indicate between-study heterogeneity of effects. Moreover, given the restricted sample sizes, a discussion of the reasons for differences in results based on study design would be too speculative.

The participants in this study were not necessarily representative of the general Danish population. This particularly applies to the participants from the *DCH* cohort, where half the study population were a selected group of body weight gainers and for participants in Inter99 study who were at high risk of ischemic heart disease. The results may therefore not be directly comparable to the general Danish population.

## Conclusion

In conclusion, our longitudinal study suggests an inverse association between alcohol intake at baseline and ΔBW and ΔWC over a period of approximately 5 years. However, this study could not demonstrate that the association between alcohol consumption and ΔBW, ΔWC or ΔWC_BMI_ depends on genetic predisposition to obesity.

## Additional files

Additional file 1: Information on the 50 SNPs included in this study. The individual SNPs are grouped according to their associated trait. (DOCX 19 kb)

Additional file 2: Interaction between each risk allele and alcohol intake (1 unit increase) in relation to ΔBW (kg/year). Model adjusted for baseline measure of the anthropometrical variables of interest, age, gender, height, smoking status, education, PA, menopausal status and total energy intake. (DOCX 33 kb)

Additional file 3: Interaction between each risk allele and alcohol intake (1 unit increase) in relation to ΔWC (cm/year). Model adjusted for baseline measure of the anthropometrical variables of interest, age, gender, height, smoking status, education, PA, menopausal status and total energy intake. (DOCX 28 kb)

Additional file 4: Interaction between each risk allele and alcohol intake (1 unit increase) in relation to ΔWC_BMI_ (cm/year). Model adjusted for baseline measure of the anthropometrical variables of interest, age, BMI, gender, height, smoking status, education, PA, menopausal status and total energy intake. (DOCX 28 kb)

Additional file 5: Annual change in BW, WC and WC_BMI_ per 1 alcohol unit/day increase in alcohol intake. (DOCX 14 kb)

Additional file 6: SNP score × alcohol interactions in relation to annual change in BW, WC and WC_BMI_. (DOCX 16 kb)

Additional file 7: Annual change in BW, WC and WC_BMI_ per 1 alcohol unit/day increase in alcohol intake. (DOCX 14 kb)

Additional file 8: SNP score × alcohol interactions in relation to annual change in BW, WC and WC_BMI_. (DOCX 15 kb)

## Abbreviations

BMI: Body mass index
BW: Body weight
CI: Confidence interval
DCH: Diet, Cancer and Health cohort
FFQ: Food frequency questionnaires
g: Gram
GWAS: Genome-wide association studies
PA: Physical activity
SNP: Single nucleotide polymorphisms
WC: Waist circumference
WC_BMI_: Waist circumference adjusted for Body Mass Index
WHR: Waist-Hip-Ratio
Yrs: Years
