# Supplementary material for: Alcohol consumption and its interaction with adiposity-associated genetic variants in relation to subsequent changes in waist circumference and body weight
Source: Nutr J. 2017 Aug 25;16:51. doi: 10.1186/s12937-017-0274-1 (PMC5574083; doi:10.1186/s12937-017-0274-1)
Supplement: Supplementary file 1 — Information on the 50 SNPs included in this study. The individual SNPs are grouped according to their associated trait. (DOCX 19 kb) [file 12937_2017_274_MOESM1_ESM.docx]

| **Additional file** **1: Information on the 50 SNPs included in this study^1^.** | | | | |  |
| --- | --- | --- | --- | --- | --- |
| **Trait** | **SNP** | **Nearest gene** | **Risk allele** | **Other allele** | |
| BMI | rs10508503 | PTER | C | T | |
| BMI | rs10838738 | MTCH2 | G | A | |
| BMI | rs10938397 | GNPDA2 | G | A | |
| BMI | rs10968576 | LRRN6C | G | A | |
| BMI | rs11847697 | PRKD1 | T | C | |
| BMI | rs12444979 | GPRC5B | C | T | |
| BMI | rs13107325 | SLC39A8 | T | C | |
| BMI | rs1424233 | MAF | A | G | |
| BMI | rs1514175 | TNNI3K | T | C | |
| BMI | rs1555543 | PTBP2 | C | A | |
| BMI | rs17782313 | MC4R | C | T | |
| BMI | rs1805081 | NPC1 | A | G | |
| BMI | rs206936 | NUDT3 | G | A | |
| BMI | rs2112347 | FLJ35779 | T | G | |
| BMI | rs2241423 | MAP2K5 | G | A | |
| BMI | rs2287019 | QPCTL | C | T | |
| BMI | rs2568958 | NEGR1 | A | G | |
| BMI | rs2890652 | LRP1B | C | T | |
| BMI | rs29941 | KCTD15 | G | A | |
| BMI | rs3810291 | TMEM160 | A | G | |
| BMI | rs4712652 | LINC00340 | A | G | |
| BMI | rs4771122 | MTIF3 | G | A | |
| BMI | rs4929949 | RPL27A | C | T | |
| BMI | rs543874 | SEC16B | G | A | |
| BMI | rs6013029 | CTNNBL1 | T | G | |
| BMI | rs6232 | PCSK1 | G | A | |
| BMI | rs6602024 | PFKP | A | G | |
| BMI | rs713586 | RBJ | C | T | |
| BMI | rs7647305 | SFRS10 | C | T | |
| BMI | rs9939609 | FTO | A | T | |
| BMI/WC | rs10146997 | NRXN3 | G | A | |
| BMI/WC | rs1121980 | FTO | A | G | |
| BMI/WC | rs7138803 | FAIM2 | A | G | |
| WC | rs12970134 | MC4R | A | G | |
| WC | rs545854 | MSRA | G | C | |
| WC | rs987237 | TFAPB2 | G | A | |
| WHR_BMI_ | rs1011731 | DNM3-PIGC | C | T | |
| WHR_BMI_ | rs10195252 | GRB14 | T | C | |
| WHR_BMI_ | rs1055144 | NFE2L3 | A | G | |
| WHR_BMI_ | rs1294421 | LY86 | G | T | |
| WHR_BMI_ | rs1443512 | HOXC13 | A | C | |
| WHR_BMI_ | rs2605100 | LYPLAL1 | G | A | |
| WHR_BMI_ | rs4823006 | ZNRF3-KREMEN1 | A | G | |
| WHR_BMI_ | rs6784615 | NISCH-STAB1 | T | C | |
| WHR_BMI_ | rs6795735 | ADAMTS9 | C | T | |
| WHR_BMI_ | rs6861681 | CPEB4 | A | G | |
| WHR_BMI_ | rs6905288 | VEGFA | A | G | |
| WHR_BMI_ | rs718314 | ITPR2-SSPN | C | T | |
| WHR_BMI_ | rs9491696 | RSPO3 | G | C | |
| WHR_BMI_ | rs984222 | TBX15-WARS2 | G | C | |
| *^1^The individual SNPs are grouped according to their associated trait.*  *Abbreviations: Body Mass Index (BMI). Waist Circumference (WC). Waist-Hip Ratio (WHR).* | | | | | |
