# Supplementary material for: Alcohol consumption and its interaction with adiposity-associated genetic variants in relation to subsequent changes in waist circumference and body weight
Source: Nutr J. 2017 Aug 25;16:51. doi: 10.1186/s12937-017-0274-1 (PMC5574083; doi:10.1186/s12937-017-0274-1)
Supplement: Supplementary file 2 — Interaction between each risk allele and alcohol intake (1 unit increase) in relation to ΔBW (kg/year). Model adjusted for baseline measure of the anthropometrical variables of interest, age, gender, height, smoking status, education, PA, menopausal status and total energy intake. (DOCX 33 kb) [file 12937_2017_274_MOESM2_ESM.docx]

| **Additional file** **2: Interaction between each risk allele and alcohol intake (1 unit increase) in relation to ΔBW (kg/year)^1^.** | | | | | | | | | | | | | | |
| --- | --- | --- | --- | --- | --- | --- | --- | --- | --- | --- | --- | --- | --- | --- |
| **Trait** | **SNP** | **MONICA** | | | **DCH** | | | **Inter99** | | | **All** | | | |
|  |  | **N** | **β** | **P** | **N** | **β** | **P** | **N** | **β** | **P** | **N** | **β** | **P** | **P_Adjusted_** |
| BMI | rs10508503 | 1233 | -0.03 | 0.41 | 1878 | 0.00 | 0.97 | 3529 | 0.00 | 0.86 | 6640 | -0.01 | 0.57 | 1 |
| BMI | rs10838738 | 1240 | 0.02 | 0.26 | 1866 | 0.01 | 0.68 | 3499 | -0.04 | 0.03 | 6605 | -0.01 | 0.54 | 1 |
| BMI | rs10938397 | 1235 | 0.00 | 0.88 | 1871 | -0.01 | 0.59 | 3477 | -0.02 | 0.27 | 6583 | -0.01 | 0.33 | 1 |
| BMI | rs10968576 | 1244 | -0.01 | 0.69 | 1867 | 0.00 | 0.99 | 3461 | 0.00 | 0.99 | 6572 | 0.00 | 0.86 | 1 |
| BMI | rs11847697 | 1245 | 0.01 | 0.90 | 1880 | 0.06 | 0.25 | 3490 | -0.02 | 0.40 | 6615 | 0.00 | 0.89 | 1 |
| BMI | rs12444979 | 1238 | -0.04 | 0.19 | 1872 | -0.04 | 0.14 | 3490 | 0.02 | 0.47 | 6600 | -0.01 | 0.37 | 1 |
| BMI | rs13107325 | 1247 | -0.03 | 0.55 | 1869 | 0.03 | 0.53 | 3485 | 0.01 | 0.76 | 6601 | 0.01 | 0.78 | 1 |
| BMI | rs1424233 | 1229 | 0.00 | 0.84 | 1877 | -0.01 | 0.67 | 3496 | -0.01 | 0.51 | 6602 | -0.01 | 0.56 | 1 |
| BMI | rs1514175 | 1242 | 0.00 | 0.89 | 1871 | 0.01 | 0.51 | 3491 | -0.02 | 0.15 | 6604 | -0.01 | 0.50 | 1 |
| BMI | rs1555543 | 1249 | -0.01 | 0.57 | 1873 | 0.04 | 0.02 | 3466 | -0.03 | 0.08 | 6588 | 0.00 | 0.82 | 1 |
| BMI | rs17782313 | 1242 | -0.04 | 0.09 | 1880 | 0.01 | 0.56 | 3512 | -0.01 | 0.56 | 6634 | -0.01 | 0.38 | 1 |
| BMI | rs1805081 | 1230 | 0.00 | 0.85 | 1873 | 0.02 | 0.30 | 3521 | 0.00 | 0.94 | 6624 | 0.01 | 0.62 | 1 |
| BMI | rs206936 | 1248 | -0.02 | 0.39 | 1881 | -0.01 | 0.80 | 3450 | -0.03 | 0.17 | 6579 | -0.02 | 0.14 | 1 |
| BMI | rs2112347 | 1234 | 0.04 | 0.10 | 1870 | -0.01 | 0.74 | 3503 | -0.02 | 0.13 | 6607 | 0.00 | 0.66 | 1 |
| BMI | rs2241423 | 1236 | 0.08 | 0.00 | 1873 | 0.01 | 0.70 | 3474 | 0.02 | 0.34 | 6583 | 0.03 | 0.01 | 0.71 |
| BMI | rs2287019 | 1242 | -0.01 | 0.68 | 1858 | 0.01 | 0.69 | 3435 | -0.02 | 0.24 | 6535 | -0.01 | 0.42 | 1 |
| BMI | rs2568958 | 1238 | -0.06 | 0.01 | 1879 | 0.02 | 0.33 | 3208 | 0.02 | 0.15 | 6325 | 0.00 | 0.96 | 1 |
| BMI | rs2890652 | 1250 | -0.01 | 0.79 | 1865 | -0.04 | 0.09 | 3494 | -0.02 | 0.43 | 6609 | -0.02 | 0.11 | 1 |
| BMI | rs29941 | 1235 | -0.01 | 0.66 | 1871 | 0.01 | 0.58 | 3211 | -0.02 | 0.19 | 6317 | -0.01 | 0.42 | 1 |
| BMI | rs3810291 | 1231 | 0.02 | 0.44 | 1878 | 0.02 | 0.31 | 3445 | 0.02 | 0.28 | 6554 | 0.02 | 0.10 | 1 |
| BMI | rs4712652 | 1230 | 0.02 | 0.21 | 1850 | 0.00 | 0.86 | 3492 | 0.01 | 0.72 | 6572 | 0.01 | 0.32 | 1 |
| BMI | rs4771122 | 1233 | 0.01 | 0.69 | 1848 | 0.02 | 0.26 | 3463 | -0.01 | 0.45 | 6544 | 0.00 | 0.76 | 1 |
| BMI | rs4929949 | 1244 | -0.01 | 0.65 | 1849 | 0.01 | 0.65 | 3491 | 0.00 | 0.96 | 6584 | 0.00 | 0.96 | 1 |
| BMI | rs543874 | 1246 | 0.00 | 0.95 | 1870 | -0.05 | 0.03 | 3211 | 0.01 | 0.79 | 6327 | -0.01 | 0.32 | 1 |
| BMI | rs6013029 | 1241 | -0.04 | 0.36 | 1873 | 0.01 | 0.79 | 3525 | 0.04 | 0.25 | 6639 | 0.01 | 0.65 | 1 |
| BMI | rs6232 | 1238 | -0.02 | 0.67 | 1877 | -0.06 | 0.11 | 3500 | 0.04 | 0.26 | 6615 | -0.01 | 0.66 | 1 |
| BMI | rs6602024 | 1239 | -0.04 | 0.20 | 1875 | -0.03 | 0.26 | 3495 | 0.04 | 0.13 | 6609 | -0.01 | 0.71 | 1 |
| BMI | rs713586 | 1249 | -0.02 | 0.46 | 1874 | 0.03 | 0.15 | 3458 | -0.01 | 0.66 | 6581 | 0.00 | 0.88 | 1 |
| BMI | rs7647305 | 1236 | -0.01 | 0.64 | 1863 | -0.03 | 0.24 | 3211 | 0.00 | 0.85 | 6310 | -0.01 | 0.31 | 1 |
| BMI | rs9939609 | 1240 | -0.02 | 0.19 | 2165 | 0.00 | 0.80 | 3459 | 0.01 | 0.63 | 6864 | -0.01 | 0.57 | 1 |
| BMI/WC | rs10146997 | 1252 | 0.09 | 0.00 | 1871 | 0.02 | 0.28 | 3501 | -0.02 | 0.26 | 6624 | 0.02 | 0.08 | 1 |
| BMI/WC | rs1121980 | 1243 | -0.02 | 0.29 | 1880 | -0.01 | 0.71 | 3209 | 0.00 | 0.99 | 6332 | -0.01 | 0.43 | 1 |
| BMI/WC | rs7138803 | 1242 | 0.04 | 0.05 | 1873 | 0.00 | 0.88 | 3209 | -0.03 | 0.10 | 6324 | 0.00 | 0.95 | 1 |
| WC | rs12970134 | 1227 | 0.01 | 0.79 | 1864 | 0.00 | 0.87 | 3494 | -0.01 | 0.74 | 6585 | 0.00 | 1.00 | 1 |
| WC | rs545854 | 1245 | -0.02 | 0.37 | 1872 | -0.01 | 0.82 | 3514 | -0.03 | 0.16 | 6631 | -0.02 | 0.13 | 1 |
| WC | rs987237 | 1243 | 0.00 | 0.86 | 1882 | 0.01 | 0.64 | 3486 | 0.03 | 0.20 | 6611 | 0.02 | 0.23 | 1 |
| WHR | rs1011731 | 1245 | -0.03 | 0.19 | 1816 | -0.06 | 0.00 | 3464 | 0.01 | 0.63 | 6525 | -0.02 | 0.06 | 1 |
| WHR | rs10195252 | 1237 | 0.04 | 0.05 | 1856 | -0.01 | 0.57 | 3464 | -0.01 | 0.37 | 6557 | 0.00 | 0.95 | 1 |
| WHR | rs1055144 | 1244 | 0.10 | 0.00 | 1875 | -0.04 | 0.17 | 3479 | 0.01 | 0.51 | 6598 | 0.02 | 0.13 | 1 |
| WHR | rs1294421 | 1250 | -0.01 | 0.60 | 1884 | 0.00 | 0.85 | 3460 | 0.01 | 0.66 | 6594 | 0.00 | 0.90 | 1 |
| WHR | rs1443512 | 1249 | -0.03 | 0.30 | 1886 | -0.04 | 0.13 | 3454 | 0.02 | 0.31 | 6589 | -0.01 | 0.53 | 1 |
| WHR | rs2605100 | 1249 | 0.02 | 0.30 | 1871 | 0.00 | 0.95 | 3492 | 0.01 | 0.77 | 6612 | 0.01 | 0.49 | 1 |
| WHR | rs4823006 | 1245 | 0.02 | 0.27 | 1874 | 0.02 | 0.37 | 3455 | 0.01 | 0.58 | 6574 | 0.02 | 0.15 | 1 |
| WHR | rs6784615 | 1252 | -0.04 | 0.34 | 1877 | 0.02 | 0.71 | 3494 | -0.01 | 0.81 | 6623 | -0.01 | 0.65 | 1 |
| WHR | rs6795735 | 1235 | 0.02 | 0.25 | 1850 | 0.01 | 0.72 | 3486 | 0.01 | 0.41 | 6571 | 0.01 | 0.18 | 1 |
| WHR | rs6861681 | 1236 | 0.04 | 0.11 | 1849 | -0.01 | 0.49 | 3481 | -0.01 | 0.46 | 6566 | 0.00 | 0.87 | 1 |
| WHR | rs6905288 | 1238 | -0.01 | 0.50 | 1879 | 0.02 | 0.25 | 3475 | 0.01 | 0.73 | 6592 | 0.01 | 0.60 | 1 |
| WHR | rs718314 | 1234 | -0.02 | 0.42 | 1862 | 0.00 | 0.99 | 3485 | -0.02 | 0.26 | 6581 | -0.01 | 0.24 | 1 |
| WHR | rs9491696 | 1239 | 0.01 | 0.62 | 1864 | 0.00 | 0.86 | 3425 | 0.02 | 0.19 | 6528 | 0.01 | 0.30 | 1 |
| WHR | rs984222 | 1246 | -0.04 | 0.03 | 1879 | -0.02 | 0.46 | 3475 | 0.01 | 0.48 | 6600 | -0.01 | 0.27 | 1 |

*^1^ Model adjusted for baseline measure of the anthropometrical variables of interest, age, gender, height, smoking status, education, PA, menopausal status and total energy intake.*
