# Supplementary material for: Alcohol consumption and its interaction with adiposity-associated genetic variants in relation to subsequent changes in waist circumference and body weight
Source: Nutr J. 2017 Aug 25;16:51. doi: 10.1186/s12937-017-0274-1 (PMC5574083; doi:10.1186/s12937-017-0274-1)
Supplement: Supplementary file 3 — Interaction between each risk allele and alcohol intake (1 unit increase) in relation to ΔWC (cm/year). Model adjusted for baseline measure of the anthropometrical variables of interest, age, gender, height, smoking status, education, PA, menopausal status and total energy intake. (DOCX 28 kb) [file 12937_2017_274_MOESM3_ESM.docx]

| **Additional file** **3: Interaction between each risk allele and alcohol intake (1 unit increase) in relation to ΔWC (cm/year)^1^.** | | | | | | | | | | | |
| --- | --- | --- | --- | --- | --- | --- | --- | --- | --- | --- | --- |
| **Trait** | **SNP** | **DCH** | | | **Inter99** | | | **All** | | | |
|  |  | **N** | **β** | **P** | **N** | **β** | **P** | **N** | **β** | **P** | **P_Adjusted_** |
| BMI | rs10508503 | 1844 | -0.01 | 0.86 | 3070 | -0.01 | 0.74 | 4914 | -0.01 | 0.71 | 1 |
| BMI | rs10838738 | 1831 | 0.02 | 0.47 | 3043 | -0.04 | 0.08 | 4874 | -0.02 | 0.35 | 1 |
| BMI | rs10938397 | 1837 | 0.02 | 0.58 | 3027 | -0.02 | 0.38 | 4864 | -0.01 | 0.67 | 1 |
| BMI | rs10968576 | 1832 | 0.01 | 0.77 | 3011 | -0.01 | 0.66 | 4843 | 0.00 | 0.86 | 1 |
| BMI | rs11847697 | 1845 | -0.04 | 0.54 | 3037 | 0.02 | 0.65 | 4882 | 0.00 | 0.89 | 1 |
| BMI | rs12444979 | 1837 | 0.02 | 0.55 | 3039 | 0.02 | 0.44 | 4876 | 0.02 | 0.33 | 1 |
| BMI | rs13107325 | 1834 | 0.04 | 0.48 | 3032 | 0.03 | 0.60 | 4866 | 0.04 | 0.38 | 1 |
| BMI | rs1424233 | 1842 | 0.04 | 0.22 | 3038 | -0.02 | 0.35 | 4880 | 0.00 | 0.93 | 1 |
| BMI | rs1514175 | 1838 | 0.01 | 0.60 | 3037 | -0.02 | 0.25 | 4875 | -0.01 | 0.54 | 1 |
| BMI | rs1555543 | 1838 | 0.04 | 0.16 | 3012 | -0.03 | 0.18 | 4850 | 0.00 | 0.79 | 1 |
| BMI | rs17782313 | 1845 | -0.03 | 0.33 | 3057 | -0.01 | 0.52 | 4902 | -0.02 | 0.27 | 1 |
| BMI | rs1805081 | 1838 | 0.00 | 0.87 | 3062 | -0.01 | 0.45 | 4900 | -0.01 | 0.59 | 1 |
| BMI | rs206936 | 1846 | 0.00 | 0.96 | 2999 | -0.04 | 0.10 | 4845 | -0.03 | 0.18 | 1 |
| BMI | rs2112347 | 1835 | 0.00 | 0.90 | 3046 | -0.03 | 0.08 | 4881 | -0.02 | 0.16 | 1 |
| BMI | rs2241423 | 1838 | 0.01 | 0.76 | 3025 | -0.01 | 0.53 | 4863 | -0.01 | 0.74 | 1 |
| BMI | rs2287019 | 1823 | 0.02 | 0.47 | 2990 | -0.02 | 0.39 | 4813 | -0.01 | 0.75 | 1 |
| BMI | rs2568958 | 1844 | -0.01 | 0.82 | 2778 | 0.01 | 0.61 | 4622 | 0.00 | 0.77 | 1 |
| BMI | rs2890652 | 1830 | -0.06 | 0.09 | 3039 | 0.00 | 0.87 | 4869 | -0.02 | 0.25 | 1 |
| BMI | rs29941 | 1836 | 0.01 | 0.75 | 2780 | -0.02 | 0.42 | 4616 | -0.01 | 0.64 | 1 |
| BMI | rs3810291 | 1843 | 0.01 | 0.84 | 2996 | 0.01 | 0.58 | 4839 | 0.01 | 0.57 | 1 |
| BMI | rs4712652 | 1816 | 0.06 | 0.03 | 3032 | 0.00 | 0.92 | 4848 | 0.02 | 0.24 | 1 |
| BMI | rs4771122 | 1813 | 0.00 | 0.98 | 3018 | -0.02 | 0.34 | 4831 | -0.01 | 0.44 | 1 |
| BMI | rs4929949 | 1815 | 0.03 | 0.31 | 3037 | -0.01 | 0.78 | 4852 | 0.01 | 0.73 | 1 |
| BMI | rs543874 | 1836 | -0.03 | 0.37 | 2780 | 0.01 | 0.57 | 4616 | 0.00 | 0.95 | 1 |
| BMI | rs6013029 | 1838 | -0.03 | 0.68 | 3071 | 0.07 | 0.12 | 4909 | 0.04 | 0.27 | 1 |
| BMI | rs6232 | 1843 | -0.03 | 0.53 | 3047 | 0.03 | 0.50 | 4890 | 0.00 | 0.89 | 1 |
| BMI | rs6602024 | 1840 | -0.03 | 0.38 | 3046 | 0.02 | 0.61 | 4886 | 0.00 | 0.87 | 1 |
| BMI | rs713586 | 1839 | 0.03 | 0.22 | 3005 | -0.02 | 0.23 | 4844 | 0.00 | 0.83 | 1 |
| BMI | rs7647305 | 1830 | -0.03 | 0.33 | 2780 | -0.02 | 0.36 | 4610 | -0.03 | 0.19 | 1 |
| BMI | rs9939609 | 2126 | -0.02 | 0.38 | 3007 | -0.01 | 0.74 | 5133 | -0.01 | 0.42 | 1 |
| BMI/WC | rs10146997 | 1837 | 0.00 | 0.91 | 3045 | 0.00 | 0.86 | 4882 | 0.00 | 0.83 | 1 |
| BMI/WC | rs1121980 | 1846 | -0.02 | 0.45 | 2778 | -0.02 | 0.42 | 4624 | -0.02 | 0.27 | 1 |
| BMI/WC | rs7138803 | 1839 | -0.01 | 0.79 | 2779 | -0.04 | 0.09 | 4618 | -0.02 | 0.13 | 1 |
| WC | rs12970134 | 1829 | -0.04 | 0.22 | 3039 | 0.01 | 0.73 | 4868 | -0.01 | 0.67 | 1 |
| WC | rs545854 | 1837 | -0.01 | 0.75 | 3060 | 0.03 | 0.34 | 4897 | 0.01 | 0.58 | 1 |
| WC | rs987237 | 1849 | 0.02 | 0.55 | 3037 | 0.02 | 0.36 | 4886 | 0.02 | 0.28 | 1 |
| WHR | rs1011731 | 1782 | -0.05 | 0.08 | 3012 | 0.01 | 0.53 | 4794 | -0.01 | 0.63 | 1 |
| WHR | rs10195252 | 1821 | 0.04 | 0.17 | 3014 | -0.01 | 0.56 | 4835 | 0.01 | 0.75 | 1 |
| WHR | rs1055144 | 1841 | -0.04 | 0.28 | 3023 | 0.02 | 0.56 | 4864 | 0.00 | 0.90 | 1 |
| WHR | rs1294421 | 1849 | 0.02 | 0.52 | 3008 | 0.04 | 0.04 | 4857 | 0.03 | 0.04 | 1 |
| WHR | rs1443512 | 1851 | -0.04 | 0.26 | 3006 | 0.03 | 0.21 | 4857 | 0.01 | 0.70 | 1 |
| WHR | rs2605100 | 1836 | 0.02 | 0.41 | 3041 | 0.00 | 0.95 | 4877 | 0.01 | 0.58 | 1 |
| WHR | rs4823006 | 1839 | 0.03 | 0.30 | 3003 | 0.03 | 0.19 | 4842 | 0.03 | 0.10 | 1 |
| WHR | rs6784615 | 1842 | -0.01 | 0.90 | 3039 | 0.00 | 0.96 | 4881 | 0.00 | 0.96 | 1 |
| WHR | rs6795735 | 1816 | 0.01 | 0.70 | 3036 | 0.01 | 0.53 | 4852 | 0.01 | 0.46 | 1 |
| WHR | rs6861681 | 1815 | -0.02 | 0.40 | 3026 | 0.02 | 0.27 | 4841 | 0.01 | 0.72 | 1 |
| WHR | rs6905288 | 1844 | -0.01 | 0.79 | 3025 | 0.00 | 0.97 | 4869 | 0.00 | 0.90 | 1 |
| WHR | rs718314 | 1827 | 0.01 | 0.80 | 3031 | -0.01 | 0.74 | 4858 | 0.00 | 0.90 | 1 |
| WHR | rs9491696 | 1829 | 0.00 | 0.89 | 2984 | 0.03 | 0.14 | 4813 | 0.02 | 0.21 | 1 |
| WHR | rs984222 | 1844 | -0.05 | 0.09 | 3020 | 0.02 | 0.34 | 4864 | 0.00 | 0.89 | 1 |

*^1^ Model adjusted for baseline measure of the anthropometrical variables of interest, age, gender, height, smoking status, education, PA, menopausal status and total energy intake.*
