# Supplementary material for: Alcohol consumption and its interaction with adiposity-associated genetic variants in relation to subsequent changes in waist circumference and body weight
Source: Nutr J. 2017 Aug 25;16:51. doi: 10.1186/s12937-017-0274-1 (PMC5574083; doi:10.1186/s12937-017-0274-1)
Supplement: Supplementary file 5 — Annual change in BW, WC and WCBMI per 1 alcohol unit/day increase in alcohol intake. (DOCX 14 kb) [file 12937_2017_274_MOESM5_ESM.docx]

| **Additional file** **5: Annual change in BW, WC and WC_BMI_ per 1 alcohol unit/day increase in alcohol intake^1^.** | | | | |
| --- | --- | --- | --- | --- |
| **Outcome** | **n** | **β** | **95% CI** | **P** |
| ΔBW | 7028 | -18.18 | -33.68, -2.67 | 0.022 |
| ΔWC | 5265 | -0.32 | -0.56, -0.09 | 0.007 |
| ΔWC_BMI_ | 5265 | -0.02 | -0.19, 0.16 | 0.860 |
| *^1^No adjustment for baseline measure of outcome.*  *Results presented in g/year and mm/year, respectively. Model adjusted for age, gender, height, smoking status, education, physical activity, menopausal status and total energy intake.* | | | | |
