# Supplementary material for: Alcohol consumption and its interaction with adiposity-associated genetic variants in relation to subsequent changes in waist circumference and body weight
Source: Nutr J. 2017 Aug 25;16:51. doi: 10.1186/s12937-017-0274-1 (PMC5574083; doi:10.1186/s12937-017-0274-1)
Supplement: Supplementary file 6 — SNP score × alcohol interactions in relation to annual change in BW, WC and WCBMI. (DOCX 16 kb) [file 12937_2017_274_MOESM6_ESM.docx]

| **Additional file** **6: SNP score × alcohol interactions in relation to annual change in BW, WC and WC_BMI_^1^.** | | | | | |
| --- | --- | --- | --- | --- | --- |
| **Outcome** | **SNP-score** | **n** | **β** | **95% CI** | **P** |
| ΔBW | Complete | 3919 | -0.08 | -4.37, 4.22 | 0.972 |
| ΔBW | BMI | 4589 | -2.11 | -6.82, 2.60 | 0.380 |
| ΔBW | WC | 5984 | 3.64 | -6.48, 13.76 | 0.481 |
| ΔBW | WHR | 5647 | 1.00 | -5.23, 7.22 | 0.754 |
| ΔWC | Complete | 2814 | -0.01 | -0.08, 0.05 | 0.687 |
| ΔWV | BMI | 3319 | -0.08 | -0.15, -0.01 | 0.029 |
| ΔWC | WC | 4369 | -0.05 | -0.21, 0.10 | 0.505 |
| ΔWC | WHR | 4115 | 0.06 | -0.04, 0.16 | 0.218 |
| ΔWC_BMI_ | Complete | 2814 | 0.00 | -0.05, 0.05 | 0.917 |
| ΔWC_BMI_ | BMI | 3319 | -0.05 | -0.10, 0.00 | 0.075 |
| ΔWC_BMI_ | WC | 4369 | -0.03 | -0.14, 0.09 | 0.638 |
| ΔWC_BMI_ | WHR | 4115 | 0.05 | -0.01, 0.12 | 0.124 |
| ^1^*No adjustment for baseline measure of outcome*  *Results presented in g/year and mm/year, respectively, and relate to the interaction (effect-modification) effects per additional risk allele for each alcohol unit/day.  Model adjusted for age, gender, height, smoking status, education, physical activity, menopausal status and total energy intake.* | | | | | |
