# Supplementary material for: Alcohol consumption and its interaction with adiposity-associated genetic variants in relation to subsequent changes in waist circumference and body weight
Source: Nutr J. 2017 Aug 25;16:51. doi: 10.1186/s12937-017-0274-1 (PMC5574083; doi:10.1186/s12937-017-0274-1)
Supplement: Supplementary file 7 — Annual change in BW, WC and WCBMI per 1 alcohol unit/day increase in alcohol intake. (DOCX 14 kb) [file 12937_2017_274_MOESM7_ESM.docx]

| **Additional file** **7: Annual change in BW, WC and WC_BMI_ per 1 alcohol unit/day increase in alcohol intake^1^.** | | | | |
| --- | --- | --- | --- | --- |
| **Outcome** | **n** | **β** | **95% CI** | **P** |
| ΔBW | 6247 | -20.1 | -36.22, -3.98 | 0.015 |
| ΔWC | 4853 | -0.02 | -0.48, 0.01 | 0.058 |
| ΔWC_BMI_ | 4853 | 0.01 | -0.04, 0.30 | 0.136 |
| *^1^Restricted on stable smokers.*  *Results presented in g/year and mm/year, respectively. Model adjusted for baseline measure of outcome, age, gender, height, smoking status, education, physical activity, menopausal status and total energy intake.* | | | | |
