# Supplementary material for: Alcohol consumption and its interaction with adiposity-associated genetic variants in relation to subsequent changes in waist circumference and body weight
Source: Nutr J. 2017 Aug 25;16:51. doi: 10.1186/s12937-017-0274-1 (PMC5574083; doi:10.1186/s12937-017-0274-1)
Supplement: Supplementary file 8 — SNP score × alcohol interactions in relation to annual change in BW, WC and WCBMI. (DOCX 15 kb) [file 12937_2017_274_MOESM8_ESM.docx]

| **Additional file** **8: SNP score × alcohol interactions in relation to annual change in BW, WC and WC_BMI_^1^.** | | | | | |
| --- | --- | --- | --- | --- | --- |
| **Outcome** | **SNP-score** | **n** | **β** | **95% CI** | **P** |
| ΔBW | Complete | 3467 | -0.86 | -5.26, 3.54 | 0.701 |
| ΔBW | BMI | 4073 | -3.44 | -8.34, 1.46 | 0.169 |
| ΔBW | WC | 5303 | 0.20 | -10.29, 10.69 | 0.970 |
| ΔBW | WHR | 4987 | 1.71 | -4.71, 8.12 | 0.602 |
| ΔWC | Complete | 2607 | -0.01 | -0.07, 0.06 | 0.837 |
| ΔWV | BMI | 3077 | -0.07 | -0.14, 0.00 | 0.058 |
| ΔWC | WC | 4036 | -0.06 | -0.22, 0.10 | 0.448 |
| ΔWC | WHR | 3784 | 0.08 | -0.02, 0.17 | 0.118 |
| ΔWC_BMI_ | Complete | 2607 | 0.01 | -0.03, 0.06 | 0.528 |
| ΔWC_BMI_ | BMI | 3077 | -0.02 | -0.07, 0.04 | 0.561 |
| ΔWC_BMI_ | WC | 4036 | -0.01 | -0.12, 0.10 | 0.904 |
| ΔWC_BMI_ | WHR | 3784 | 0.05 | -0.01, 0.12 | 0.109 |
| *^1^Restricted on stable smokers.*  *Results presented in g/year and mm/year, respectively, and relate to the interaction (effect-modification) effects per additional risk allele for each alcohol unit/day.  Model adjusted for baseline measure of outcome age, gender, height, smoking status, education, physical activity, menopausal status and total energy intake.* | | | | | |
